# Cardioprotective effect of epigallocatechin gallate in myocardial ischemia/reperfusion injury and myocardial infarction: a meta-analysis in preclinical animal studies

**DOI:** 10.1038/s41598-023-41275-2

**Published:** 2023-08-28

**Authors:** Xin-Yu Wei, Yi-Fan Zeng, Qi-Hao Guo, Ji-Jia Liu, Ni Yin, Yan Liu, Wen-Jing Zeng

**Affiliations:** 1 Department of Pharmacy, Xiangya Hospital, Central South University, Changsha, Hunan China; 2 National Clinical Research Center for Geriatric Disorders, Xiangya Hospital, Central South University, Changsha, China; 3 Department of Cardiovascular Surgery, The Second Xiangya Hospital, Central South University, Changsha, Hunan China; 4 Department of Pharmacy, Shengjing Hospital, China Medical University, Shenyang, China; 5 Department of Pharmacy, Hunan Aerospace Hospital, Hunan Normal University, Changsha, Hunan China

**Keywords:** Preclinical research, Myocardial infarction

## Abstract

This meta-analysis aims to determine the efficacy of Epigallocatechin gallate (EGCG) in the treatment of myocardial ischemia–reperfusion injury (MIRI) and summarize the mechanisms involved. Literature from six databases including Web of Science, PubMed, Embase, China National Knowledge Infrastructure (CNKI), Wan-Fang database, and VIP database (VIP) were systematically searched. All the analysis were conducted by R. Twenty-five eligible studies involving 443 animals were included in this meta-analysis. The results indicated that compared to controls, EGCG exerts a cardioprotective effect by reducing myocardial infarct size (SMD = −4.06; 95% CI: −5.17, −2.94; P < 0.01; I^2^ = 77%). The funnel plot revealed publication bias. Moreover, EGCG significantly improves cardiac function, serum myocardial injury enzyme, and oxidative stress levels in MIRI animal models. This meta-analysis demonstrates that EGCG exhibits therapeutic promise in animal models of MIRI. However, further validation is still needed in large animal models and large clinical studies.

## Introduction

Despite substantial advances in the prevention and treatment of cardiovascular diseases (CVD), it is still the major cause of global mortality, of which the toll of death due to ischemic heart disease accounts for approximately 50%^1–3^. Acute myocardial infarction (AMI) is usually caused by prolonged ischemia of the myocardial cell^4^. The reperfusion treatment strategies for AMI are to open the occluded artery by thrombolytic therapy or percutaneous coronary intervention (PCI) or coronary artery bypass grafting^5–7^. Although early diagnosis and timely reperfusion are the most effective strategies to reduce myocardial ischemia injury and myocardial infarction size, reperfusion itself can also lead to myocardial cell injury and death, which is called myocardial ischemia/reperfusion injury (MIRI)^8^. The manifestations of MIRI mainly include ventricular arrhythmias, myocardial stunning (myocardial systolic dysfunction), microvascular obstruction (MVO), and lethal myocardial reperfusion injury^9^, and its pathological processes include oxidative stress, cytosolic calcium overload, rapid restoration of intracellular pH, mitochondrial dysfunction, cell apoptosis, inflammation^9,10^. Although numerous explorations have been made on the treatment of MIRI in recent years, no effective drug therapy has been found. Therefore, there is an urgent need to seek new effective therapeutic strategies to prevent and treat MIRI**.**

Green tea has been one of the most popular beverages worldwide for thousands of years, playing a pivotal role in the prevention and remission of cancer, cardiovascular disease, diabetes, and other diseases^11,12^. Epigallocatechin gallate (EGCG), also known as epigallocatechin-3-O-gallate, is the main component of green tea polyphenols. Previous studies have reported that EGCG confers a promising cardiovascular protective effect against cardiovascular diseases, especially MIRI, by reducing the production of reactive oxygen species (ROS), preventing intracellular Ca^2+^ overload^13–15^, inhibiting the inflammatory response and cell apoptosis, and promoting free radical scavenging. In addition, it can also alleviate atherosclerosis, heart failure, heart hypertrophy, arrhythmia, and other cardiovascular diseases. However, the specific efficacy and determine its underlying molecular mechanisms of EGCG in treating MIRI have not been systematically assessed to date. Therefore, we performed a meta-analysis to evaluate the cardioprotective efficacy and potential mechanisms of EGCG in MIRI animal models.

## Methods

This meta-analysis has been registered in PROSPERO (ID: CRD42023390971).

## Search strategy

We performed a systematic literature search on Web of Science, PubMed, Embase, China National Knowledge Infrastructure (CNKI), the Wan-Fang database, and the VIP database between database inception and January 2023 without language restrictions. Search items include: "myocardial I/R", "myocardial I/R injury”, “myocardial ischemia–reperfusion injury”, “myocardial ischemia–reperfusion”, “epigallocatechin-3-O-gallate”, “epigallocatechin gallate”, “epigallocatechin-3-gallate”, and “EGCG”.

### Study selection

Studies that met all of the following pre-established criteria were included: (1) animal models of MIRI were established by ligating the left anterior descending (LAD) coronary artery or injecting intravenously vasoconstrictor; (2) EGCG was the only intervention with a control group receiving placebo fluid or no treatment; (3) in vivo or ex vivo animal studies; (4) the primary outcome measures were myocardial infarction size, cardiac function parameters, serum myocardial enzyme and oxidative stress markers and the secondary outcome measures were mechanisms of EGCG to protect against MIRI. The pre-established exclusion criteria were as follows: (1) abstracts or meeting posters; (2) no detailed data was provided; (3) no animal model; (4) without a control group; (5) in vitro studies; (6) EGCG is not the only intervention.

### Data extraction

Two researchers independently filtrated the title, abstract, and full text, then extracted the details of the study. When any discrepancy arose, they negotiated with correspondence authors. The research details extracted include: (1) Author information: author, publication year, and country; (2) Animal information: species, sex, and weight; (3) Animal models: anesthetic, model methods, and the number of each group; (4) Drug administration: method, dosage, and duration of administration; (5) Outcome record: the mean and standard deviation of the primary (infarct size) and secondary outcomes (cardiac function, serum myocardial injury enzyme, and oxidative stress levels). When different doses of the drug were used, data in the highest dose group was extracted. When there were multiple time points in the reperfusion time, only the last time point was recorded.

### Quality assessment

The two researchers used the SYRCLE’s RoB tool to assess and score the quality of the included studies on a 10-item scale of one point for each item, as follows: peer-reviewed publication, control of temperature, random allocation to treatment or control, blinded induction of the model, blinded evaluation of the outcome, appropriate use of anesthetic, appropriate animal model, sample size calculation, compliance with animal welfare regulations, statement of potential conflict of interests. Divergences were resolved by discussing with the corresponding author.

### Statistical analysis

All statistical data in this meta-analysis were analyzed using R software (Version 4.2.2). The standard mean difference (SMD) and the 95% confidence interval (CI) were used to evaluate the effect of EGCG on myocardial infarct size, cardiac function parameters, serum myocardial enzyme, and oxidative stress markers. Heterogeneity across studies was assessed by the Cochran test and I^2^ statistics. It is generally believed that I^2^ values of 0–25%, 25–75%, and 75–100% correspond to low heterogeneity, medium heterogeneity, and high heterogeneity, respectively. The random-effects model was used to estimate the overall effect without considering heterogeneity due to different animal populations of animal-based meta-analysis. Publication bias was analyzed by funnel plots and Egger's test when more than ten studies were included in the meta-analysis. Sensitivity analysis was used to explore sources of heterogeneity. The probability values of P-value less than 0.05 are considered statistically significant.

## Results

### Study selection

**Figure 1:**
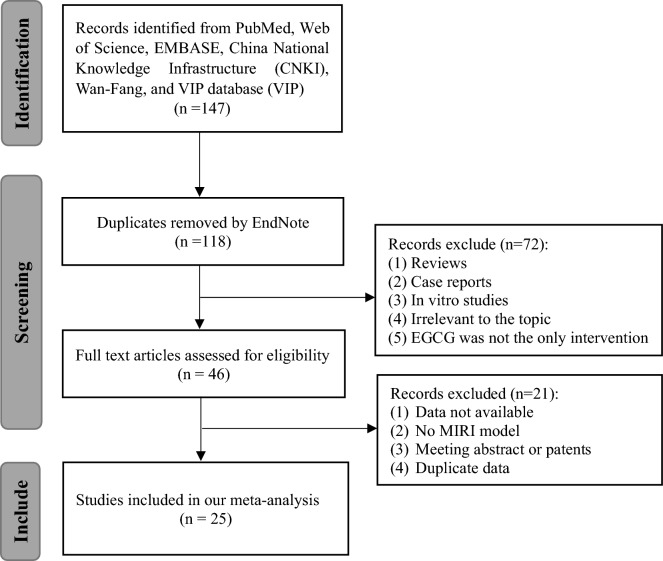
Flowchart for database search and study selection.

A total of 147 potential records were retrieved from six databases, of which 29 articles were removed for reduplication. After screening titles and abstracts, 72 studies were discarded for reasons including (1) reviews, (2) case reports, (3) in vitro studies, (4) irrelevant to the topic, and (5) EGCG was not the only intervention. The full text of the remaining studies was examined, of which 21 were excluded for the following reasons: (1) no available data, (2) no MIRI model, (3) meeting abstracts or patents, and (4) duplication. Finally, 25 eligible studies were included^3,14,16–39^. The PRISMA flow diagram of literature screening in this study is shown in Fig. 1.

### Characteristics of included studies

A total of 25 studies with 443 animals (221 in the EGCG-treated group and 222 in the control group) were included between 2004 and 2021. In terms of countries, 12 of the 26 studies were conducted in China, five studies in Korea, two each in India and Japan, and one each in the United Kingdom, the United States, Egypt, and Germany. Wistar rats were used in nine studies, SD rats were used in 12 studies, C57BL/6 mice were used in two studies, and Hartley strain guinea pigs and Chinchilla Bastard rabbits were used in one study. A total of 22 studies established the MIRI model by LAD ligation, of which eight studies were by LAD occlusion in isolated perfused hearts with Langendorff, and three studies established MIRI by intravenous injection of isoprenaline (ISO). EGCG was administered intravenously in nine studies, intraperitoneally in one study, orally in seven studies, and perfused to the heart in eight studies. The characteristics of the 25 enrolled studies are presented in Table 1.

**Table 1:** Baseline characteristics of included studies.

Author	Year	Country	Species	Weight	n (EGCG/Ctrl)	Anesthetic	I/R duration	Type of I/R	Drug delivery method	Duration	Outcome
Aneja et al.^17^	2004	United States	Wistar rats	NR	6/6	Thiopentone sodium (70 mg/kg)	30 min/2 h	In vivo	Intravenous (10 mg/kg)	1 h	CK
Devika et al.^16^	2008	India	Wistar rats	140–160 g	8/8	NR	ISO (100 mg/kg)	In vivo	Intragastric (30 mg/kg)	3 weeks	CK, CK-MB, LDH, SOD, GSH, CAT
Devika et al.^18^	2008	India	Wistar rats	140–160 g	8/8	NR	ISO (100 mg/kg)	In vivo	Intragastric (30 mg/kg)	3 weeks	SOD, GSH, CAT
Fu et al.^19^	2019	China	C57BL/6 mice	16–18 g	15/15	Isoflurane (3 ml/kg)	30 min/4 h	In vivo	Intragastric (5 mg/g)	1 week	Infarct size, SOD, MDA
Hirai et al.^14^	2007	Japan	Hartley strain guinea pigs	300–350 g	5–6/5–6	Diethyl ether	40 min/40 min	Ex vivo	Perfused (30uM)	4 min	LVEDP, LVDP
Hu et al.^20^	2005	China	Wistar rats	200–250 g	10/10	Pentobarbital sodium (30 mg/kg)	30 min/60 min	In vivo	Intravenous (20 mg/kg)	60 min	LVSP, LVEDP, dP/dt max, CK, LDH, SOD, MDA
Kim et al.^21^	2010	Korea	Wistar rats	280–330 g	8/9	Pentobarbital sodium (100 mg/kg)	30 min/2 h	Ex vivo	Perfused (10 μM)	35 min	Infarct size, LVDP, dP/dt max
Kim et al.^22^	2014	Korea	SD rats	250–300 g	6/5	Pentobarbital (100 mg/kg)	30 min/2 h	In vivo	Intravenous (10 mg/kg)	5 min	Infarct size
Lee et al.^23^	2012	Korea	SD rats	280-330 g	9/9	Pentobarbital sodium (50 mg/kg)	30 min/2 h	Ex vivo	Perfused (10 μM)	40 min	Infarct size
Li et al.^24^	2015	China	SD rats	250–280 g	8/8	NR	30 min/1 h	In vivo	Intravenous (20 mg/kg)	20 min	CK-MB
Lin et al.^25^	2020	China	Wistar rats	NR	5/5	NR	Permanent LAD ligation	In vivo	Intragastric (50 mg/kg)	4 weeks	Infarct size
Othman et al.^26^	2017	Egypt	Wistar rats	280–300 g	6/6	NR	ISO (100 mg/kg)	In vivo	Intraperitoneal (15 mg/kg)	1 week	CK-MB, LDH, TnT, SOD, MDA, SOD
Piao et al.^27^	2011	Korea	SD rats	250–280 g	12/12	Cervical dislocation	20 min/2 h	Ex vivo	Perfused (5 μM)	10 min	Infarct size, CK, LDH
Qin et al.^28^	2017	China	Wistar rats	220–250 g	10/10	Pentobarbital sodium (100 mg/kg)	30 min/2 h	In vivo	Intravenous (10 mg/kg)	5 min	Infarct size, CK, LDH
Salameh et al.^29^	2018	Germany	Chinchilla Bastard rabbits	1.5–2 kg	6/6	Medetomidine (0.2 mg/kg) and ketamine (20 mg/kg)	90 min/1 h	Ex vivo	Perfused (20 μM)	90 min	dP/dt max
Song et al.^30^	2010	Korea	Wistar rats	280–330 g	7/8	Pentobarbital sodium (100 mg/kg)	30 min/2 h	In vivo	Perfused (10 μM)	40 min	Infarct size
Townsend et al.^31^	2004	UK	SD rats	NR	8/8	NR	35 min/2 h	Ex vivo	Perfused (100 μM)	30 min	Infarct size
Tu et al.^32^	2021	China	SD rats	210–250 g	3/3	Pentobarbital sodium (50 mg/kg)	30 min/2 h	Ex vivo	Perfused (10 mg)	30 min	Infarct size, TnT
Wu et al.^33^	2017	China	SD rats	260–280 g	18/18	Pentobarbital sodium (65 mg/kg)	30 min/2 h	In vivo	Intragastric (100 mg/kg)	2 weeks	Infarct size, LVSP, dP/dt max, LDH
Xin et al.^34^	2008	China	SD rats	250–300 g	20/20	Pentobarbital sodium (30 mg/kg)	30 min/6 h	In vivo	Intravenous (20 mg/kg)	20 min	Infarct size, CK-MB
Xuan et al.^35^	2016	China	SD rats	250–280 g	8/8	20% ethyl carbamate (5 ml/kg)	30 min/2 h	In vivo	Intravenous (10 mg/kg)	10 min	Infarct size, LVSP, LVEDP, dP/dt max, CK-MB, LDH
Yanagi et al.^38^	2011	Japan	SD rats	350–450 g	12/12	Diethyl ether and pentobarbital sodium (50 mg/kg)	30 min/1 h	Ex vivo	Purfused (10 mmol/L)	2 weeks	LVDP, dP/dt max
Zeng et al.^36^	2021	China	C57BL/6 mice	25 g	10/10	Isoflurane	45 min/3 h	In vivo	Intragastric (250 mg/kg)	10 days	Infarct size
Zhang et al.^37^	2019	China	SD rats	150–200 g	6/6	10% chloral hydrate	30 min/12 h	In vivo	Intravenous (10 mg/kg)	30 min	Infarct size
Zhang et al.^39^	2019	China	SD rats	250–300 g	6/6	10% chloral hydrate	30 min/12 h	In vivo	Intravenous (20 mg/kg)	30 min	LVSP, LVEDP, dP/dt max, CK-MB, LDH

*I/R* ischemia/reperfusion, *EGCG* epigallocatechin gallate, *SD* Sprague–Dawley, *ISO* isoproterenol, *LVDP* left ventricular developed pressure, *LVEDP* left ventricular end-diastolic pressure, *LVSP* left ventricular systolic pressure, *dP/dT max* maximum 1st derivative of developed pressure, *CK* creatine kinase, *CK-MB* creatine kinase isoenzyme, *LDH* lactic dehydrogenase, *TnT* troponin T, *SOD* superoxide dismutase, *MDA* malondialdehyde, *CAT* catalase, *NR* not reported.

### The methodological quality of the included studies

The quality score of the included studies ranged from 3 to 7. Three studies scored 4 points, nine studies scored 5 points, and 11 studies scored 6 points. The remaining two studies scored 3 and 7, respectively. All studies were published in peer-reviewed journals. All animals were randomly allocated to the treatment or control groups with appropriate animal models (aged, diabetic, or hypertensive). However, no study describes the blinded induction of the model and sample size calculation. Blinded assessment of outcome was described in two studies. Nine studies mentioned control of temperature. A total of 21 studies used anesthetic without significant intrinsic cardioprotective activity. Animals in 21 studies complied with animal welfare regulations. Only five studies included a statement of potential conflict of interest. The methodological quality of the included studies is shown in Table 2. The molecular mechanism of included studies that EGCG protects cardiomyocytes from MIRI was summarized in Table 3.

**Table 2:** Quality assessment of included studies.

Author	Year	A	B	C	D	E	F	G	H	I	J	Score
Aneja et al.^17^,	2004	★	★	★			★	★		★		6
Devika et al.^16^	2008	★		★			★	★		★		5
Devika et al.^18^	2008	★		★				★		★		4
Fu et al.^19^	2019	★		★			★	★		★		5
Hirai et al.^14^	2007	★	★	★			★	★				5
Hu et al.^20^	2005	★		★			★	★				4
Kim et al.^21^	2010	★		★		★	★	★		★		6
Kim et al.^22^	2014	★	★	★			★	★		★		6
Lee et al.^23^	2012	★		★			★	★		★		5
Li et al.^24^	2015	★		★				★		★		4
Lin et al.^25^	2020	★		★			★	★		★		5
Othman et al.^26^	2017	★	★	★			★	★		★	★	7
Piao et al.^27^	2011	★	★	★				★		★		5
Qin et al.^28^	2017	★	★	★			★	★		★		6
Salameh et al.^29^	2018	★		★			★	★		★	★	6
Song et al.^30^	2010	★		★		★	★	★		★		6
Townsend et al.^31^	2004	★		★				★				3
Tu et al.^32^	2021	★		★			★	★		★		5
Wu et al.^33^	2017	★	★	★			★	★		★		6
Xin et al.^34^	2008	★		★			★	★		★		5
Xuan et al.^35^	2016	★	★	★			★	★		★		6
Yanagi et al.^38^	2011	★		★			★	★		★		5
Zeng et al.^36^	2021	★		★			★	★		★	★	6
Zhang et al.^37^	2019	★		★			★	★		★	★	6
Zhang et al.^39^	2019	★		★			★	★		★	★	6

A, peer-reviewed publication; B, control of temperature; C, random allocation to treatment or control; D, blinded induction of model; E, blinded assessment of outcome; F, appropriate use of anesthetic; G, appropriate animal model; H, sample size calculation; I, compliance with animal welfare regulations; J, statement of potential conflict of interests.

**Table 3:** Molecular and cellular mechanisms of myocardial ischemia/reperfusion injury treated with epigallocatechin gallate.

Author	Year	Proposed mechanisms
Aneja et al.^17^	2004	Inhibit IKK/NF-kB and c-Jun/AP-1 pathways
Devika et al.^16^	2008	Increase antioxidant capacity and maintain ATPase and ion levels
Devika et al.^18^	2008	Increase antioxidant capacity
Fu et al.^19^	2019	Enhanced antioxidant and anti-apoptotic capacity through inhibition of PI3K/Akt pathway
Hirai et al.^14^	2007	Relieve Ca^2+^ overload and inhibit apoptosis by reduce caspase-3 expression
Hu et al.^20^	2005	Inhibit apoptosis by increase Bcl-2 and decrease Bax expression
Kim et al.^21^	2010	–
Kim et al.^22^	2014	Promote cell survival by activation of PI3K/Akt/GSK-3βand reduce apoptosis by inhibition of P38/JNK-MAPK pathway
Lee et al.^23^	2012	Activate ADR
Li et al.^24^	2015	Inhibit of autophagy by activation of the PI3K/Akt/Beclin axis
Lin et al.^25^	2020	Promote expression of miR-145 and attenuate Dab2/Wnt3a/β-catenin pathways
Othman et al.^26^	2017	Inhibit oxidative stress by increasing SOD and CAT expression and reduce apoptosis by decreasing p53, Bax, caspase-3/9 and increasing Bcl-2 expression
Piao et al.^27^	2011	Antioxidant and anti-apoptotic
Qin et al.^28^	2017	Enhance anti-inflammation by reducing mtDNA release due to PI3K/Akt activation
Salameh et al.^29^	2018	–
Song et al.^30^	2010	Alleviates Ca^2+^ overload by Inhibition of mKATP channel opening
Townsend et al.^31^	2004	Inhibits apoptosis by suppressing STAT-1/Fas signaling pathway
Tu et al.^32^	2021	Reduce intracellular Ca^2+^ concentration, increase TnT concentration, promote NAD+ concentration, and improve the ultrastructure of cardiomyocytes
Wu et al.^33^	2017	Increase the expression of SIRT1
Xin et al.^34^	2008	Inhibits apoptosis by reducing Caspase-3 expression
Xuan et al.^35^	2016	Restores autophagic flux by activating of PI3K/Akt/mTOR pathway
Yanagi et al.^38^	2011	Reduction of 8-OHdG enhances antioxidant capacity, blocks p38 MAPK phosphorylation and reduces Caspase-3 expression to inhibit apoptosis
Zeng et al.^36^	2021	Modulate Gm4419/DUSP5/ERK1/2-mediated autophagy
Zhang et al.^37^	2019	miR-384-mediated autophagy by targeting Beclin-1 via activating the PI3K/Akt signaling pathway
Zhang et al.^39^	2019	Attenuate mitochondrial impairment and myocardial apoptosis by regulation of miR-30a/p53 axis

### Outcome measures

#### Myocardial infarct size

**Figure 2:**
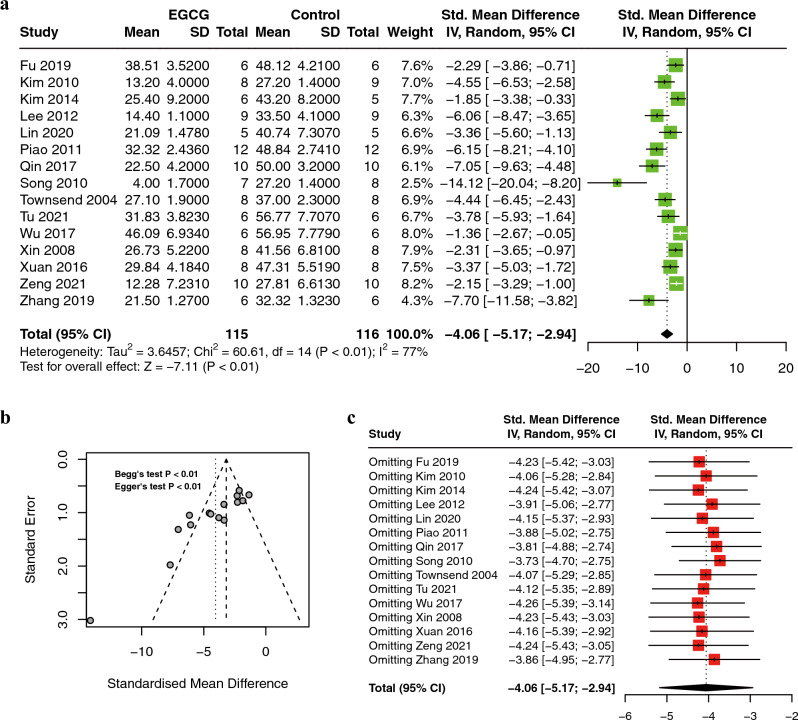
Forest plot displaying the protective effect of EGCG on infarct size in an animal model of myocardial ischemia/reperfusion injury (**A**); funnel plot assessing publication bias of infarct size in included studies (**B**); sensitivity analysis of infarct size (**C**). *EGCG* epigallocatechin gallate.

A meta-analysis of 15 studies, involving 231 animals^19,21–23,25,27,28,30–37^, showed that the EGCG treatment significantly reduced the myocardial infarct size (IS) compared to controls (SMD = −4.06; 95% CI: −5.17, −2.94; *P* < 0.01; I^2^ = 77%) (Fig. 2a). Due to the apparent heterogeneity of the included publications, we performed a funnel plot and sensitivity analysis. The funnel plot was asymmetrical and the P-value of Egger's test and Begg's test was less than 0.05, indicating publication bias **(**Fig. 2b**)**. The sensitivity analysis did not reveal any heterogeneous source of IS. This supported the result of IS was stable and reliable **(**Fig. 2c**)**.

We conducted subgroup analysis to explore the sources of heterogeneity further. As shown in Table 4, there are significant differences in the analysis between subgroups of species, drug delivery method, and duration, which may be an important source of heterogeneity.

**Table 4:** Subgroup analysis of pooled estimates of infarct size.

Subgroup	No. of studies	SMD	95% CI	P value between subgroups	heterogeneity within subgroups	
					I^2^(%)	P value
**Species**				**0.02**		
SD rats	9	− 3.79	− 5.09, − 2.50		75	< 0.01
Wistar rats	4	− 6.60	-10.48, − 2.71		78	< 0.01
C57BL/6 mice	2	− 2.19	− 3.12, − 1.27		0	0.89
**Type of I/R**				0.24		
In vivo	10	− 3.78	− 5.46, − 2.10		77	< 0.01
Ex vivo	5	− 4.94	− 5.89, − 4.00		0	0.45
**IS measure method**				0.21		
AN/LV	6	−3.26	−4.29, −2.23		54	0.06
AN/AAR	9	−4.61	−6.48, −2.74		84	< 0.01
**Drug delivery method**				**< 0.01**		
Perfused	6	− 5.28	− 6.42, − 4.14		61	0.03
Intragastric	4	− 2.06	− 2.78, − 1.35		0	0.47
Intravenous	5	− 4.09	− 6.29, − 1.88		78	< 0.01
**Duration**				**< 0.01**		
≤ 30 min	8	− 4.26	− 5.66, − 2.86		74	< 0.01
30 min to 1 day	5	− 7.59	− 12.70, − 2.47		78	< 0.01
≥ 1 day	4	− 2.06	− 2.78, − 1.35		0	0.47
**Ischemia time**				0.22		
≤ 30 min	12	− 4.40	− 5.86, − 2.94		80	< 0.01
> 30 min	3	− 3.11	− 4.55, − 1.67		77	< 0.01
**Reperfusion time**				**0.02**		
≤ 2 h	8	− 4.26	− 6.15, − 3.08		81	< 0.01
> 2 h	10	− 2.53	− 3.24, − 1.82		50	0.09

Significant values are in bold.

*SMD* standardized mean difference, *CI* confidence interval, *SD rats* Sprague–Dawley rats, *I/R* ischemia/reperfusion, *IS* infarct size, *AN* area necrosis, *LV* left ventricle, *AAR* area at risk, *ISO* isoproterenol.

#### Cardiac function

**Figure 3:**
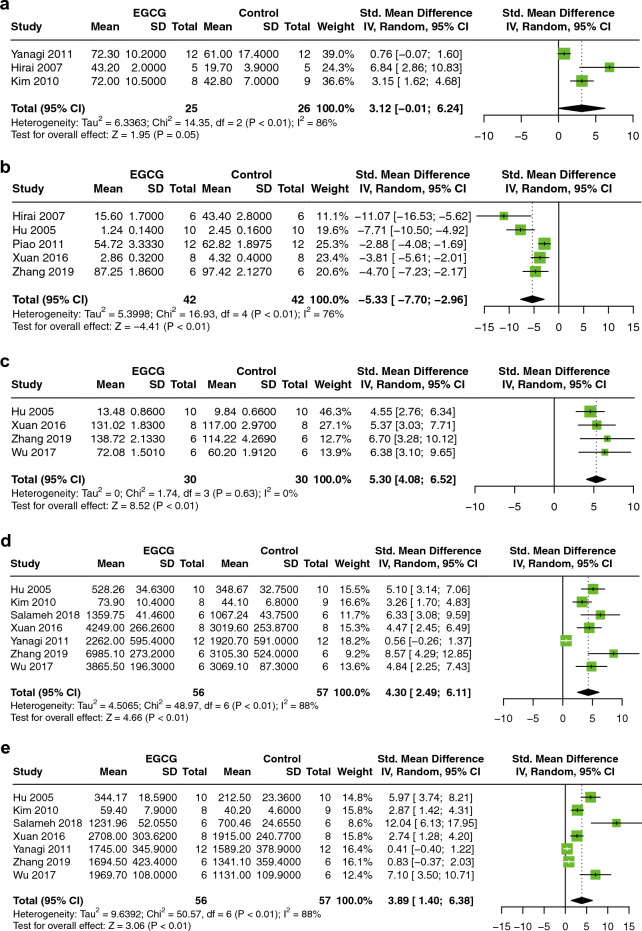
Forest plot illustrating the cardioprotective effect of EGCG on myocardial ischemia/reperfusion injury animal models of LVDP (**A**), LVEDP (**B**), LVSP (**C**), + dP/dt max (**D**), − dP/dt max (**E**). *LVDP* left ventricular developed pressure, *LVEDP* left ventricular end-diastolic pressure, *LVSP* left ventricular systolic pressure, *dP/dt max* maximum, 1st derivative of developed pressure, *EGCG* epigallocatechin gallate.

Five markers, consisting of left ventricular developed pressure (LVDP), left ventricular end-diastolic pressure (LVEDP), left ventricular systolic pressure (LVSP), + dP/dT max, and −dP/dt max, were analyzed to reveal the improvement effect of EGCG on cardiac function in MIRI animals. Meta-analysis of three studies with 51 animals indicated that compared to controls EGCG has a potential effect on decreasing LVDP (SMD = 3.12; 95% CI: −0.01, 6.24; *P* = 0.05; I^2^ = 86%; Fig. 3a)^14,21,38^. Altogether, five studies of 84 animals showed that EGCG significantly reduced LVEDP (SMD = −5.33; 95% CI: −7.70, −2.96; *P* < 0.01; I^2^ = 76%; Fig. 3b)^14,20,27,35,39^. A meta-analysis of 4 studies involving 60 animals found that EGCG marked increased LVSP (SMD = 5.30; 95% CI: 4.08, 6.52; *P* < 0.01; I^2^ = 0%; Fig. 3c)^20,33,35,39^. A total of seven studies involving 113 animals used + dP/dt max and − dP/dt max as outcome indicators, and the analysis results showed that EGCG treatment significantly improved + dP/dt max (SMD = 4.30; 95% CI: 2.49, 6.11; *P* < 0.01; I^2^ = 88%; Fig. 3d) and − dP/dt max (SMD = 3.89; 95% CI: 1.40, 6.38; *P* < 0.01; I^2^ = 88%; Fig. 3e)^20,21,29,33,35,38,39^.

#### Cardiac enzymes

**Figure 4:**
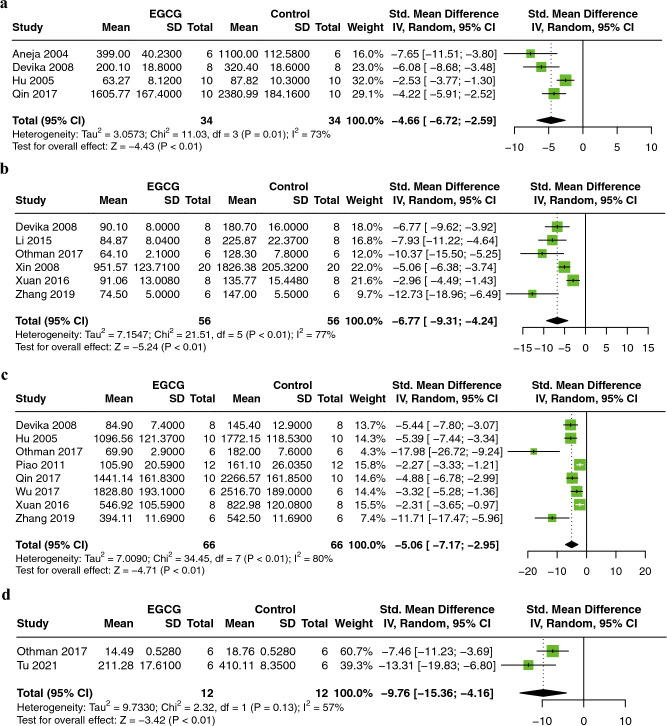
Forest plot showing cardioprotective effects of EGCG on CK (**A**), CK-MB (**B**), LDH (**C**), and TnT (**D**) in an animal model of myocardial ischemia/reperfusion injury. *CK* creatine kinase, *CK-MB* creatine kinase isoenzyme, *LDH* lactate dehydrogenase, *TnT* troponin T, *EGCG* epigallocatechin gallate.

The effects of EGCG on serum biomarkers of myocardial injury were evaluated by creatine kinase (CK), creatine kinase isoenzyme (CK-MB), lactic dehydrogenase (LDH), and troponin T (TnT). 4 publications involved 64 animals utilizing CK^16,17,20,28^, six publications involved 112 animals utilizing CK-MB^16,24,26,34,35,39^, eight publications involved 132 animals utilizing LDH^16,20,26–28,33,35,39^, and two publications involved 24 animals utilizing TnT^26,32^, as the outcome measure. Compared to controls, EGCG significantly reduces serum CK (SMD = −4.66; 95% CI: −6.72, −2.59; *P* < 0.01; I^2^ = 73%; Fig. 4a), CK-MB (SMD = −6.77; 95% CI: −9.31, −4.24; *P* < 0.01; I^2^ = 77%; Fig. 4b), LDH (SMD = −5.06; 95% CI: −7.17, −2.95; *P* < 0.01; I^2^ = 80%; Fig. 4c), and TnT (SMD = −9.76; 95% CI: −15.36, −4.16; *P* < 0.01; I^2^ = 57%; Fig. 4d) in MIRI animals.

#### Oxidative stress levels

**Figure 5:**
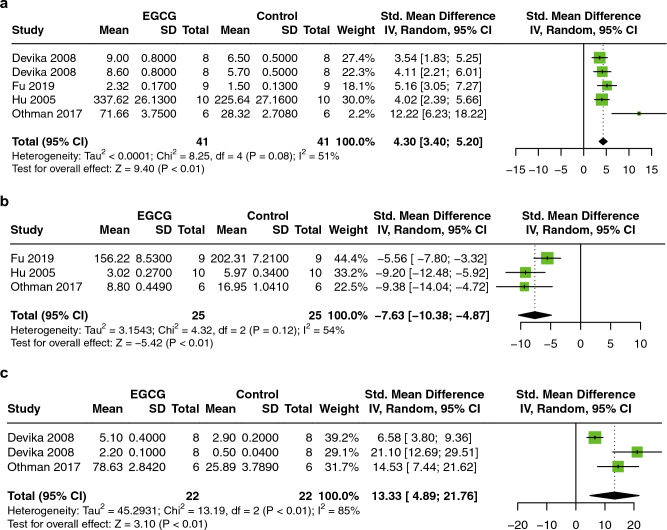
Forest plot illustrating the cardioprotective effects of EGCG on SOD (**A**), MDA (**B**), and CAT (**C**) in an animal model of myocardial ischemia/reperfusion injury. *SOD* superoxide dismutase, *MDA* malondialdehyde, *CAT* catalase.

To evaluate the antioxidant capacity of EGCG in MIRI animal models, malondialdehyde (MDA), superoxide dismutase (SOD), and catalase (CAT) were analyzed. 5 studies with a total of 82 animals for SOD^16,18–20,26^, three studies with 50 animals for MDA^19,20,26^, and three studies with 44 animals for CAT^16,18,26^ were included. As compared to controls, EGCG significantly attenuated oxidative stress by increasing serum SOD (SMD = 4.30; 95% CI: 3.40, 5.20; P < 0.01; I^2^ = 51%; Fig. 5a), decreasing MDA (SMD = −7.63; 95% CI: −10.38, −4.87; P < 0.01; I^2^ = 54%; Fig. 5b), and increasing CAT (SMD = 13.33; 95% CI: 4.89, 21.76; P < 0.01; I^2^ = 85%; Fig. 5c).

## Discussion

### Summary of evidence

We conduct the first meta-analysis of preclinical studies to assess the cardioprotective efficacy of EGCG in MIRI. A total of 25 studies with 443 animals were included. The evidence available in our study indicated that EGCG exerted a cardioprotective role by reducing myocardial IS and the expression of serum myocardial enzymes, improving cardiac function parameters, and reducing oxidative stress level.

### Molecular mechanisms

Numerous studies have shown that MIRI is closely associated with various pathological processes such as inflammation, oxidation stress, cardiomyocyte apoptosis, autophagy, etc^40^. An in-depth understanding of the underlying mechanisms of EGCG will serve to better understand the cardioprotective effects of EGCG. Therefore, we summarize the potential mechanisms involved in the myocardial protective effects of EGCG as follows:

### Anti-inflammation

During MIRI, necrotic cardiomyocytes activate inflammatory responses and exacerbate myocardial injury by inducing oxidative stress, triggering the complement cascade, and releasing danger-associated molecular patterns (DAMPs). Myeloperoxidase (MPO) is a neutrophil-specific heme peroxidase that is closely related to the inflammatory process of ischemia–reperfusion injury^41^. EGCG plays an anti-inflammatory role by reducing MPO activity and the infiltration of neutrophils^17^.

Mitochondrial DNA (mtDNA), a naked circular or linear DNA, has been identified as a damage-associated molecular pattern (DAMP) that can trigger a series of inflammatory responses^42,43^. In MIRI, the expression of mtDNA was positively correlated with the expression of TNF-a, IL-6, and IL-8^44^. The PI3K/Akt pathway reduces myocardial injury by negatively regulating the inflammatory response^45^. The activation of the PI3K/Akt pathway is a critical pathway of cardioprotective and is associated with preservation of mitochondrial function during MIRI^46^. EGCG inhibits inflammation response to reduce myocardial IS by activating the PI3K/Akt pathway to reduce the release of pro-inflammatory mediator mtDNA and the secretion of inflammatory factors in plasma^28^. In addition, EGCG exerts anti-inflammatory effects and protects cardiomyocytes by inhibiting IKK/NF-kB and c-Jun/AP-1 pathways^17^.

### Antioxidant

Oxidative stress caused by increased reactive oxygen species (ROS) is one of the main pathological mechanisms for the occurrence and development of MIRI. Due to the presence of multiple phenolic hydroxyl groups that are easily oxidized to quinones in the structure, EGCG exerts powerful antioxidant properties in the treatment of MIRI by regulating the redox system to scavenge free radicals and inhibiting lipid peroxidation, thereby alleviating myocardial damage^47,48^. Specifically, EGCG plays an antioxidant effect by significantly increasing the endogenous antioxidant activity of antioxidant enzymes (SOD, CAT, GPx, GRx, GST) and antioxidants (vitamin C, vitamin E, ceruloplasmin), and inhibiting the accumulation of MDA, thiobarbituric acid reactive substances, and uric acid^26,49^.

SIRT1 (Sirtuin 1) is a nicotinamide adenine dinucleotide (NAD)-dependent histone deacetylase involved in regulating oxidative stress resistance under ischemic and hypoxic conditions^50^. In diabetic MIRI rats, EGCG increases the expression of the antioxidant enzyme manganese superoxide dismutase (MnSOD) by activating SIRT1 and reducing oxidative stress damage^33^. Calcium overload is a major cause of cellular damage during MIRI^51^. Activation of KATP channels protects cardiomyocytes from MIRI-induced Ca^2+^ overload^52^. It has been found that the reduction in infarct size produced by myocardial ischemic preconditioning and pre-ischemic drug therapy is partly attributed to mitochondrial potassium ATP (mKATP) channels^53,54^. In addition, in Ossabaw minipigs, activating KATP channels by the ischemic preconditioning (IPC) process can delay the magnitude of ST-segment elevation^55^, further suggesting that opening mitochondrial KATP channels can play a cardioprotective role. During myocardial ischemia in isolated mice hearts, EGCG opens mKATP channels by activating A1 and A2B adenosine receptors to relieve Ca+ overload pressure and increase NAD+ expression, reduce the opening time of mitochondrial permeability transition (mPTP) pore and finally reduce the amount of ROS^14,23,30,56^.

### Anti-apoptosis

Apoptosis, as one of the critical mechanisms in the MIRI process, plays a dual role in MIRI according to the degree of apoptosis. On the one hand, proper apoptosis reduces myocardial cell necrosis and plays a protective role; on the other hand, excessive apoptosis aggravates myocardial injury and accelerates myocardial cell death.

In the MIRI model, EGCG alleviated cardiomyocyte apoptosis/exerts antiapoptotic effects by up-regulating the expression of anti-apoptotic protein Bcl-2, down-regulating the expression of pro-apoptotic proteins p53, Bax, cleaved-caspase-3 and caspase-9^26,27^. Existing evidence has identified the PI3K/Akt signaling pathway played a protective role in protecting cardiomyocytes from the two aspects of promoting the survival of cardiomyocytes and inhibiting the apoptosis of cardiomyocytes by regulating the expression of apoptotic proteins and transcription factors^57^. In MIRI models, EGCG increases the survival rate of cardiomyocytes by activating PI3K/Akt signaling pathway, promoting the phosphorylation of eNOS, and increasing the NO content^35^. JNK and p38 are members of the mitogen-activated protein kinase (MAPK) family, and the JNK/p38 MAPK cascade plays a pivotal role in mediating apoptosis. EGCG inhibits p38 and JNK phosphorylation, decreases Caspase-3 expression, plays an antiapoptotic role in cardiomyocytes, and reduces the size of myocardial infarction^22,38^. STAT-1 is a signal transducer and transcriptional activator, and its phosphorylation can induce apoptosis by mediating the expression of apoptosis-related genes^58^. EGCG is an effective inhibitor of STAT1 phosphorylation, which can reduce the expression of caspase-3 and the degree of myocardial cell apoptosis by inhibiting the activation of the STAT-1/Fas pathway^59^. miR-30, a member of the MicroRNA family, regulates cell apoptosis by targeting the inhibition of the mitochondrial apoptosis activator p53^60^. In hypoxia-reoxygenation (H/R)-induced H9C2 cells and I/R-induced rats, EGCG can inhibit mPTP opening and anti-apoptotic protein expression by activating the miR-30a/p53 signaling pathway^15^.

### Autophagy

Autophagy plays opposite roles in different periods of myocardial injury. Autophagy in ischemia can supply energy and play a role in cardiac protection, while in reperfusion its excessive activation accelerates cardiomyocyte death^61^. During ischemia, EGCG trigger autophagy to protect cells from apoptosis by regulating mTOR negative feedback mechanism^24^. During reperfusion, EGCG protects against MIRI by activating PI3K/Akt/mTOR signaling cascade, reducing Beclin-1 expression, and restoring autophagy flux to inhibit excessive autophagy^24,35^. Recent studies have shown that long non-coding RNA GM4419 can alleviate myocardial infarction by activating miR-682/TRF3 in I/R and H/R-induced myocardial injury models^62^. EGCG reduces the degree of myocardial injury by reducing Gm4419 expression and epigenetic silencing/inhibiting DUSP5/ERK1/2 signaling pathway-mediated autophagy in H_2_O_2_-induced cardiomyocytes and I/R-induced mouse models^63^. ATG4C, a cysteine enzyme, is involved in autophagy by regulating the functions of LC3 and ATG8^64,65^. EGCG can increase ATG4C expression and decrease LC3II expression, thus inhibiting H/R-induced apoptosis and autophagy of H9C2 cells^66^. In atherosclerotic diseases, miR-384 has been found to target and inhibit Beclin-1 to suppress macrophage autophagy^27^. In MIRI, EGCG can inhibit autophagy and alleviate the injury by activating PI3K/Akt pathway, increasing miR-384, and decreasing Beclin1^37^. EGCG can exert cardioprotective effects from three aspects of anti-inflammation, anti-apoptosis, and autophagy by regulating the PI3K/Akt pathway, suggesting an important role of the PI3K/Akt pathway in the cardioprotective process of EGCG.

### Antiplatelet effect

During myocardial infarction, platelets play a dual role of promoting arterial thrombosis leading to cardiac injury and regulating cardiomyocyte secretion of factors leading to cardioprotection^67,68^. It has been found that EGCG can inhibit platelet aggregation induced by U46619, collagen, arachidonic acid, and toxic carotenoids and shear force-induced platelet adhesion dose-dependently by suppressing PLCγ2 and tyrosine phosphorylation of various platelet proteins, up-regulating the expression of intracellular PGD2, blocking the increase of intracytoplasmic free calcium ions and reducing the release of arachidonic acid (AA), thus delaying the formation of arterial thrombus and exerting potent antithrombotic effects. What’s more, its combination with common antiplatelet therapeutic agents, aspirin (ASA), clopidogrel (CPD), and tiglitazarol (TCG), did not further inhibit platelet aggregation resulting in bleeding complications, demonstrating the potent antiplatelet effect of EGCG and its favorable safety profile. The antiplatelet and thrombotic activities of EGCG may be partially attributed to the presence of the galloyl group at the 3' position of C ring^69–71^. In addition, EGCG inhibits platelet activation by inhibiting microsomal cyclooxygenase-1 activity in platelets as well as platelet extracellular vesicle release^72,73^. During MIRI, platelets can exacerbate ischemia/reperfusion (IR) injury by promoting thrombosis, decreasing myocardial perfusion, secreting vasoconstrictors, and causing endothelial dysfunction^67,68^. Therefore, we speculated that EGCG may exert cardioprotective effects during MIRI by inhibiting platelet aggregation and thrombosis, but further experimental validation is needed.

### Coronary microcirculation

Myocardium ischemia–reperfusion inevitably leads to myocardial cell and coronary microvascular injury. Among them, coronary microvascular injury includes various mechanisms such as coronary microembolism (CME), platelet activation, endothelial dysfunction, and increased permeability, which eventually lead to capillary injury, complications of no-reflow, intramyocardial hemorrhage, and adverse microvascular obstruction, which has been identified as one of the key factors affecting the prognosis of patients with acute myocardial infarction^74–76^. Coronary microcirculation has increasingly become an effective target for cardioprotection during the treatment of acute myocardial infarction. Cardiomyocyte apoptosis and myocardial inflammation induced by coronary microembolism, a common complication during ACS and PCI treatment, are considered to be one of the main mechanisms of myocardial injury and cardiac dysfunction^77^. Inhibition of cardiomyocyte apoptosis and myocardial inflammation attenuated CME-induced myocardial injury and improved cardiac dysfunction^78^. Although there is no report of EGCG improving coronary microcirculation by treating coronary microembolism, other flavonoids such as curcumin (CCM) have been shown to inhibit CME-induced myocardial inflammation and cardiomyocyte apoptosis through the TLR4/MyD88/NF-kβ signaling axis^79^, implying that EGCG may also have the potential of improving coronary microcirculation, but the specific research still needs to be further studied. In addition, a study based on four different structures of theaflavins (TFs) found that chemicals containing only a single galloyl group improved coronary microcirculation, while none or two galloyl groups chemicals were ineffective^80^. Since the chemical structure of EGCG happens to contain a single galloyl group, we speculate that EGCG may also have the effect of improving coronary microcirculation. In addition, two clinical trials have found that EGCG supplementation improves endothelial dysfunction in the short term during the treatment of patients with coronary artery disease^81^. Therefore, we speculated that EGCG might also have the capability to improve coronary microcirculation according to both the effects of its structural analogs as well as its structural properties, but it remains to be further verified experimentally.

### Clinical trial

Although there is currently a dearth of clinical trials on the cardioprotective effects of EGCG against MIRI, several clinical trial studies have reported that EGCG protects against multiple aspects of a wide range of cardiovascular diseases. For example, two clinical studies found that EGCG reduced the risk of cardiovascular disease in obese subjects by significantly lowering plasma triglyceride levels, as well as blood pressure^82,83^. In normal male healthy volunteers, EGCG may delay the progression of oxidative stress-associated atherosclerotic disease by decreasing low-density lipoprotein (LDL) oxidizing capacity^84^. A clinical trial based on patients with mild to moderate hypertension also discovered that the administration of Benifuuki, whose active ingredient is an EGCG-O-methylated derivative, exerts a hypotensive effect by significantly inhibiting angiotensin I-converting enzyme activity^85^. Furthermore, in patients with amyloid transthyretin cardiomyopathy, EGCG may curb cardiac amyloidosis by reducing left ventricular myocardial mass^86,87^. In terms of anti-platelet drug therapy, EGCG was found to reduce adenosine diphosphate (ADP)- and collagen (COL)-induced platelet aggregation, as well as shear force-induced platelet adhesion dose-dependently, and its combination with common antiplatelet therapeutic agents, aspirin (ASA), clopidogrel (CPD), and tiglitazarol (TCG), did not further inhibit platelet aggregation resulting in bleeding complications, demonstrating the potent antiplatelet effect of EGCG and its favorable safety profile^71^. As for the effects of EGCG on endothelial dysfunction, clinical trials based on healthy subjects have shown that EGCG has no effect on improving endothelial dysfunction^88^; whereas, in patients with atherosclerosis, EGCG can reduce the incidence of cardiovascular disease by improving endothelial function^89^; and in patients with coronary artery disease, the acute administration of EGCG significantly reversed endothelial dysfunction in coronary patients in the short term. The ability of EGCG to improve endothelial dysfunction may be related to the subjects studied in the clinical trials, and EGCG may be effective in improving endothelial dysfunction in patients with cardiovascular disease in the short term, but not in healthy subjects, and the specific efficacy of EGCG remains to be further verified by trials in the future. The above clinical trial results suggest that EGCG may exert cardioprotective effects through multiple mechanisms, including lowering blood pressure, improving endothelial dysfunction, and inhibiting platelet aggregation and adhesion.

### Implications

Reperfusion injury will inevitably lead to myocardial cell death and cardiac dysfunction during the treatment of acute myocardial infarction^90^, but until now there is no clinical intervention drug to improve MIRI^91^, so it is urgent to seek a new potential therapeutic drug to improve MIRI. Studies reported that EGCG reduces myocardial injury by reducing oxidative stress, inhibiting apoptosis and inflammatory response, and regulating autophagy and mitochondrial function during MIRI^47^. The results of this study showed that EGCG significantly reduces myocardial infarct size, improves cardiac function, down-regulates myocardial enzyme levels, and inhibits oxidative stress to play a cardioprotective role in MIRI animal models.

Studies have shown that meta-analysis and systematic review to evaluate the therapeutic efficacy of drugs for experimental animal research can help the translation of research results from animal experiments to clinical applications and narrow the gap between the two^92^. During the study inclusion process, we considered the relevance of the article to the research topic during the title and abstract selection process and the full-text review process and evaluated the quality of the article according to 10 criteria. These steps contributed to improving the stability and reliability of the results of this meta-analysis. However, the results of this study only used youthful and healthy small animal models and did not include aged small animal models with multiple coexisting diseases such as diabetes and hypertension as well as coadministration of medications, which may differ from the complex pathology of patients in the actual clinical setting. According to Improving the Preclinical Assessment of Cardioprotective Therapies (IMPACT) guidelines in the EU Cardiac Protection Cost Action Guidelines, the first step in reducing the risk of failure in the translation of preclinical research into clinical research requires the use of healthy, young animals for initial experiments, but in the future, small animal models with multiple confounding factors still need to be further verified^93^. This study only included young and healthy small animal models, so the actual efficacy of EGCG still needs to be further verified in small animal models with at least one confounding factor. In addition, the results of this study were not validated in large animal models, which are crucial for the translation of preclinical research to clinical research, because the anatomy and vascular dynamics of large animals, especially pigs, are much closer to the actual configuration of human beings so that the clinical model can better mimic that of MIRI under specific conditions, which can aide in clinical translation. Moreover, animal research itself has certain methodological flaws, design variations, and publication bias caused by the fact that negative results are more difficult to be published, further widening the gap between animal experiments and clinical applications^94^. Nevertheless, to further facilitate the translation of EGCG from animal studies to clinical practice, more small animal models with multiple confounding factors, high-quality large-scale animal studies, and randomized controlled trials are still needed for further discussion and validation.

### Limitations

First, we only retrieved studies in Chinese and English databases, lacking studies in other language databases, which may cause a certain degree of selection bias. Secondly, negative results from drug studies are less likely to be published, which may lead to an overestimation of drug efficacy. Thirdly, all the animals included in the study did not adopt the disease model of myocardial injury and related comorbidities, such as advanced age, hypertension, hyperlipidemia, diabetes, etc., while patients with clinical myocardial injury often suffer from multiple diseases.

## Conclusions

This meta-analysis demonstrates that EGCG exhibits therapeutic promise in animal models of MIRI. However, further validation is still needed in large animal models and large clinical studies.

## Data Availability

The data used to support the findings of this study are included in the article. Further inquiries can be directed to the corresponding authors.

## Abbreviations

EGCG: Epigallocatechin gallate
MIRI: Myocardial ischemia–reperfusion injury
CVD: Cardiovascular diseases
AMI: Acute myocardial infarction
PCI: Percutaneous coronary intervention
MVO: Microvascular obstruction
ROS: Reactive oxygen species
LAD: Left anterior descending
SMD: Standard mean difference
CI: Confidence interval
ISO: Isoprenaline
IS: Infarct size
LVDP: Left ventricular developed pressure
LVEDP: Left ventricular end-diastolic pressure
LVSP: Left ventricular systolic pressure
CK: Creatine kinase
CK-MB: Creatine kinase isoenzyme
LDH: Lactic dehydrogenase
TnT: Troponin T
MDA: Malondialdehyde
COL: Collagen
SOD: Superoxide dismutase
CAT: Catalase
DAMPs: Danger-associated molecular patterns
MPO: Myeloperoxidase
mtDNA: Mitochondrial DNA
GPx: Glutathione peroxidase
GRx: Glutaredoxi
GST: Glutathione *S*-transferase
NAD: Nicotinamide adenine dinucleotide
mKATP: Mitochondrial potassium ATP
IPC: Ischemic preconditioning
mPTP: Mitochondrial permeability transition
MAPK: Mitogen-activated protein kinase
H/R: Hypoxia-reoxygenation
AA: Arachidonic acid
ASA: Aspirin
CPD: Clopidogrel
TCG: Tiglitazarol
CME: Coronary microembolism
LDL: Low density lipoprotein
ATTR: Amyloid transthyretin
